# Use and utility of endocrine multidisciplinary tumour board: an appraisal from a tertiary centre

**DOI:** 10.3389/fendo.2025.1513893

**Published:** 2025-05-27

**Authors:** Clotilde Sparano, Letizia Canu, Giuliano Perigli, Roberto Santoro, Silvia Pradella, Giulia Grazzini, Monica Mangoni, Gabriele Simontacchi, Benedetta Fibbi, Vania Vezzosi, Catia Olianti, Mario Maggi, Luisa Petrone

**Affiliations:** ^1^ Department of Experimental and Clinical Biomedical Sciences “Mario Serio”, University of Florence, Florence, Italy; ^2^ Endocrinology Unit, Careggi University Hospital, Florence, Italy; ^3^ Centro di Ricerca e Innovazione sulle Patologie Surrenaliche, Azienda Ospedaliero-Universitaria (AOU) Careggi, Florence, Italy; ^4^ ENS@T Center of Excellence, University of Florence, Florence, Italy; ^5^ Department of Experimental and Clinical Medicine, University of Florence, Florence, Italy; ^6^ Head and Neck Oncology and Robotic Surgery, Department of Experimental and Clinical Medicine, University of Florence, Florence, Italy; ^7^ Department of Radiology, Careggi University Hospital, Florence, Italy; ^8^ Radiotherapy Unit, Department of Experimental, Clinical and Biomedical Sciences, University of Florence, Florence, Italy; ^9^ Endocrinology Unit, Medical-Geriatric Department, Careggi University Hospital, Florence, Italy; ^10^ Department of Histopathology and Molecular Diagnostics, Careggi University Hospital, Florence, Italy; ^11^ Unit of Nuclear Medicine, Department of Image Diagnostics, Careggi University Hospital, Florence, Italy

**Keywords:** multidisciplinary tumour board, endocrine neoplasms, thyroid neoplasms, health care, rare diseases

## Abstract

**Introduction:**

The Endocrine Multidisciplinary Tumour Board (EMTB) is a specialised board for endocrine tumours, including thyroid, adrenal, and rare endocrine neoplasms. Although required by major guidelines, little is known about the current EMTB composition and working outcomes. The present study aims to analyse the use and support provided by an experienced EMTB, highlighting the skills of this board.

**Methods:**

This monocentric and retrospective study considered all the cases discussed (N=1038, concerning 835 patients) within the ETMB of Careggi University Hospital of Florence from January 1st, 2021, to December 31st, 2023. The queries have been standardised into five major groups. Besides treatment and follow-up indications, particular attention has been paid to the need for repeated discussions, additional indications, imaging revisions, and overall survival (OS) outcomes.

**Results:**

Thyroid and rare cancers were the most frequently represented (64% and 32%, respectively). At logistic regression analysis, the need for multiple discussions was associated with being a rare disease (p<0.001), familiar syndrome (p=0.003), or adrenal masses (p=0.005). When the query was "imaging review," external imaging was more often re-evaluated (p=0.027) due to differing results at EMTB revision, and in about 51% of these cases, further insights were requested. Compared to external control groups, Anaplastic Thyroid Carcinoma and Adrenocortical Carcinoma showed improved OS, 7.84 vs 2.46 months (p=0.049) and 51.92 vs 26.17 months (p=0.0076), respectively. From the hormonal perspective, further hormonal investigations were required in about 16% of eligible cases.

**Conclusions:**

EMTB is pivotal in managing and optimising common and rare endocrine tumour workups.

## 1 Introduction

The last decades have witnessed deep changes in cancer management. Among these developments, the rise of the multidisciplinary tumour boards (MTB) represents one of the main clinical progresses in the oncological setting. The possibility of meeting with several specialised physicians to discuss the same disease criticisms at once has shifted patients' care from an autarchic model to a collegial and all-rounded standard.

The conventional backbone of MTB requires specific and highly skilled core members, i.e., oncologists, surgeons, pathologists, radiologists, and radiotherapists. Besides, several other physicians may attend the meetings to outline patients' history and related disease queries.

As expected, *big killer* cancers, such as breast, lung, or pancreatic ones, are the first and most known model of MTB, and they have also been proven to improve various disease outcomes, including survival (1–4). On the sidelines, many other oncological and non-oncological conditions drew inspiration from the expertise enhancement of multidisciplinary discussions, and several boards have progressively developed.

Based on the above, Tuscany and, in particular, Florence Careggi University Hospital has carried out a gradual refinement process of the oncological offer, including the rise of several MTBs.

The Careggi Endocrine Multidisciplinary Tumour Board (EMTB) was founded in 2014, and this board is one of the cutting-edge committees of this strand. The term "endocrine tumours" encompasses several different cancers affecting different glands (i.e., thyroid, parathyroid, adrenal, and neuroendocrine neoplasms), each disclosing specific peculiarities and management modalities. On the one hand, most of these tumours are slow-growing and long-surviving, at risk of eventual chronic oncologic disease. On the other hand, a proportion of patients suffer from rare endocrine tumours or require particular care due to hormonal secretions, notably before or during invasive proceedings. Furthermore, a variable share of these patients discloses a genetic predisposition to multiple endocrine cancers, which means regular and differing screenings. Finally, the treatment goals of these tumours often diverge from those of traditional oncology, which usually focuses on survival outcomes. These features have led to formal rule adjustments, where the endocrinologist became indispensable as a core member, avoiding the systematic need for a general oncologist on this board (according to the regional legislative decree n. 155, February 2^nd,^ 2006).

The Careggi EMTB has evolved significantly over the years, and although the value of multidisciplinary meetings is recognised, the actual usefulness of EMTB discussions has yet to be assessed. The structure and functions of the board exhibit considerable heterogeneity across the nation, hindering meaningful comparisons among different centres. In this context, the current study aims to describe the functions and clinical support provided by a trained EMTB from a tertiary and experienced centre. As a secondary goal, to explore the potential benefits regarding survival in eligible carcinomas, we also analysed survival outcomes for two very rare and highly aggressive endocrine tumours, namely Anaplastic Thyroid Carcinoma (ATC) and Adrenocortical Carcinoma (ACC). Finally, we aimed to systematise the queries and outcomes of the discussions to promote reproducibility and future studies, paving the way for potential standardisation while enhancing the unique features of the specific board.

## 2 Materials and methods

This study considered all the EMTB-discussed cases from January 1^st^, 2021 to December 31^st^, 2023. Eligible data regarded thyroid, parathyroid, adrenal, and neuroendocrine diseases (thoracic and gastroenteropancreatic [GEP] neuroendocrine neoplasm [NEN]). Rare cancers correspond to the international definition of cancer with an incidence of <6 cases/100000 people and include rare familiar and sporadic thyroid, parathyroid, adrenal tumours, and NEN as listed in the RARECARE project (5). Pituitary adenomas and other suprasellar neoplasms are discussed monthly in separate meetings, including neurosurgeons and neuro-interventional radiologists; therefore, they are not included in the present study.

Ethics committee approval of this study was waived due to the teamwork survey design, which was based on physicians' clinical practice from an anonymous storage EMTB dataset. Considering the survival outcome, patients' data were collected according to the Italian Thyroid Cancer Observatory (protocol code N°11016) and ENS@T registries (protocol code N°59/11, version 1.3).

### 2.1 Endocrine multidisciplinary tumour board: composition and skills

In agreement with the regional legislative decree n. 155 of February 2^nd^, 2006, the Careggi University Hospital EMTB consists of a group of specific physicians - named core members - and one coordinator.

The EMTB core members comprise at least one delegate in each category: endocrinologist, surgeon, radiologist, pathologist, radiation oncologist, and nuclear medicine physician. Oncologists attend the meeting on request. Core members require a proven and long-lasting experience in endocrine tumour management in their field. Other specialists, such as cardiologists or paediatricians, may join the meeting whenever necessary.

The EMTB coordinator is an experienced endocrinologist, and his role encompasses several functions, including meeting planning and organisation, management of case presentations, annual institutional reports, and clinical and scientific updates.

All the meetings take place weekly in a fully equipped room, with two large screens to collegially review radiological or scintigraphy images. The internal network guarantees full access to radiological data.

During each meeting, a reference host is charged with the presentation of his patients' cases. Case hosts may be the referral endocrinologists or any other physicians who need collegial discussion for specific queries during disease management.

### 2.2 Queries and report

Since 2021, patients' reports have been computerized, producing an electronic form available on the scheduled date. Each reference host has to fulfil a standardized report summarising the main disease features of the cases, including patient features, biochemical and radiological diagnostics, eventual previous treatments, and the discussion query. Five categories have been set up to standardise the main case queries. **Table 1** summarises the main discussion category and its specific definition to appropriately classify each patient.

**Table 1:** Definition and main features of each query category.

Query Category	Definition and Eligibility	Case Hosts
Histological Confirmation (HC)	HC means discussion of new histological results related to any endocrine carcinoma of interest (thyroid, parathyroid, adrenal, NEN). HR includes: -the assessment of potential clinical and histological risk factors -the need for specific insights -indications to FU modalities	Usually, the first specialist who receives the histological report - i.e., surgeons - presents the case to the EMTB.
Imaging Review (IR)	IR means the revision of specific radiological or nuclear medicine imaging^a:^ -general image revisions or specific imaging comparison -mutual matching between radiological and nuclear medicine imaging -presurgical revision of imaging may be requested by surgeons. Patients who perform radiological imaging outside of our Institution may be asked to consign their radiological supports to perform internal revision.	Any host may present the case.
Management advice (MA)	MA means case discussions about clinical or biochemical patients' issues, e.g.: -advice about potential specialistic or molecular assessments. -second-level hormonal tests or further radiological imaging according to the case. -an eventual treatment strategy	Reference host or other host may present the case.
Recurrence suspect (RS)	RS deals with: -patients with suspected recurrent or relapsed disease -cases with discordant or doubtful findings.	Reference host or other specialist may present the case.
Surgery proposal (SP)	SP means: -collegial discussion of surgical indication. -modality and extension of the surgery may be discussed, along with potential combined surgery (for instance, cases with concurrent thyroid and adrenal surgical indication, in eligible patients). -eventual presurgical insights may be requested by surgeons or endocrinologists.	Both surgeons and reference hosts may propose surgery.

^a^radiological imaging includes: computer tomography (CT), Magnetic Resonance (MRI); Scintigraphy imaging includes: 18-fluorodeoxyglucose (18FDG-PET), 68 Gallium-DOTA- (68G-PET), 6-18F-fluoro-L-3,4-dihydroxyphenylalanine (18F-FDOPA) positron emission tomography/computed tomography scan (PET/TC), post-radioiodine treatment whole body scan (post-RAI WB scan).

NEN, neuroendocrine neoplasms; FU, follow-up; EMTB, Endocrine Multidisciplinary Tumour Board.

All patients of surgical interest were often discussed as pre- and post-surgical cases. However, in the event of thyroid cancers, few cases usually require pre-surgical consultations, and, considering the internal long-standing working group, only patients with known criticisms (i.e., concurrent serious medical condition, extent of surgery for indeterminate cytology, or small medullary thyroid carcinomas) are currently discussed.

After each discussion, an official patient report is produced with the EMTB's indications, core-member signature, and additional present physicians' by-lines.

### 2.3 Additional EMTB indications

Each EMTB report provides specific conclusions and indications. Besides follow-up and therapeutic indications, EMTB may also suggest eventual diagnostic insights. In particular, according to the case features, eventual further histological, imaging, nuclear medicine, or hormonal investigation may be proposed.

Considering histological insights, eligible cases may benefit from histological re-challenge by biopsy, tissue-slide revision, or additional histological processes.

Nuclear medicine insights include any scintigraphy imaging among 18-fluorodeoxyglucose (^18^FDG-PET), 68 Gallium-DOTA- (^68^G-PET), 18F-fluoro-L-3,4-dihydroxyphenylalanine (^18^F-FDOPA) positron emission tomography/computed tomography scan (PET/TC), ^123^Iodine metaiodobenzylguanidine (MIBG), whole-body (WB) 131-Iodine scintigraphy and SPECT-CT. Radiological insights include second-level diagnostics, such as special Magnetic Resonance (MRI) studies (for instance, whole-body MRI) or specific execution modalities for computer tomography (CT) scans (for instance, contrast medium wash-out sequences for adrenal studies). Finally, complex cases may require specific physician clinical evaluation, including both expert endocrinology physicians or other specialists, according to cases.

Somatic or germinal molecular testing may also be suggested.

From the hormonal perspective, a share of thyroid, adrenal, and neuroendocrine tumours require screening for hormonal secretion. The majority of patients usually perform the hormonal screening during the outpatient evaluation and are discussed when all the hormonal features are available. External patients or cases that don't yet have a reference endocrinologist may require specific indications in that regard. Hormonal insights include evaluation of secretion, specific hormonal preparation before invasive procedures or surgery, and the need for provocative diagnostic tests.

### 2.4 Statistical analysis

Continuous variables have been expressed as the mean ± standard deviation or median [interquartile range] when normally or non-normally distributed, respectively. Categorical variables have been expressed as numbers and percentages. T-student or Mann-Whitney tests have been applied to assess differences in normally or non-normally distributed continuous variables, respectively. Chi-square tests have been used to compare categorical variables. Multivariate analysis by logistic regressions was used to further verify significant associations. Survival analysis by Kaplan-Meier curves (6, 7) has been performed on two very rare, highly aggressive tumours, i.e., Anaplastic Thyroid Carcinoma (ATC) and Adrenocortical Carcinoma (ACC). Data for both tumours have been obtained from our EMTB register for cases. For ATC controls, data were obtained from the database of The Cancer Genome Atlas Program (TGCA) as published on the website (https://www.cbioportal.org/study/summary?id=thyroid_gatci_2024) (8–11) and selected by being at the bottom of the insertion list in the database on February 25^th,^2025, in a ratio of one to one and matched by cases' age. For ACC controls, we followed the same criteria (https://www.cbioportal.org/study/summary?id=acc_tcga_pan_can_atlas_2018) (8) but matched also for the initial disease-stage of cases. Patients' survival has been calculated from diagnosis to eventual death or the last available information. All the analyses have been performed with IBM SPSS version 28.0. All the figures are original, and they have been created with Microsoft^®^ Excel^®^, GraphPad Prism version 9.0 for Windows, GraphPad Software, Boston, Massachusetts USA, www.graphpad.com or Jamovi (12)

## 3 Results

### 3.1 General overview

From January 1^st^, 2021, to December 31^st^, 2023, 1038 cases concerning 835 patients were discussed within the EMTB. Considering the clinical features of the population, most of the patients were female (n=560, 67.1%), and the mean age was 55 ± 15.7 years old. When considering gender prevalence separately in the thyroid, adrenal, and rare disease (familiar and sporadic) groups, female gender was more often represented only within the thyroid category (76.1%, p<0.001), while genders were equally distributed in adrenal and rare disease categories. Patients with thyroid carcinoma were younger than those having an adrenal mass (p<0.001) or a rare disease (p=0.005), with a mean age of 52.42 ± 15.68 vs. 62.66 ± 12.65 and 56.24 ± 15.67, respectively. However, no differences in terms of age were found comparing rare vs. non-rare diseases (56.24 ± 15.67 vs. 54.66 ± 15.67, respectively, p=0.210).

The number of discussed cases increased from 297 (2021) to 442 (2023) per year. **Table 2** shows the number and typology of the discussed cases over three years, along with the number of individual patients evaluated. Please note that individual cases could be discussed from once to several times. As expected, thyroid cancers were the most represented (n= 538, 64.4%), and, therefore, their evaluations were the most frequent (n= 604), followed by adrenal masses (n evaluation=296, relative to n= 204 patients). Familiar tumours concerning 60 patients were evaluated 106 times (10.2% of the whole sample). The most frequent familiar tumours were Pheochromocytoma and Paragangliomas syndromes type 1, 3, and 4 (52.9%), followed by Multiple Neuroendocrine Neoplasms 1 and 2 A/B (27.9%) and a motley group of other very rare syndromes (19.2%) including Von Hippel Lindau and Neurofibromatosis type 1. However, when considering all rare diseases (familiar and sporadic), they were evaluated 332 times (32% of the whole sample) and were relative to 204 patients. At univariate analysis, rare diseases most often required repeated EMTB discussions (range of item discussion from 1 to 10), compared to non-rare diseases (range of item discussion from 1 to 4) (p<0.001). Indeed, at logistic regression analysis, when adjusted for age, gender, and the main categories of cases (i.e., thyroid, adrenal, rare, and familiar diseases), the need for multiple discussions (dummy variable: no/yes) was independently and positively associated with the condition of being a rare disease [odds ratio (OR)=3.59, 95CI: 2.13-6.05, p<0.001], being a familiar syndrome (OR=2.99, 95CI: 1.44-6.22, p=0.003) or concerning an adrenal mass (OR=2.60, 95CI:1.34-5.03, p=0.005) (**Table 3**).

**Table 2:** Overview of the number of evaluated cases according to type, rarity, and genetic outcome.

			Sporadic		Familiar		Total for disease type and rarity	
Type	Subtype	Category	#patients for each category	#Discussions for each category	#patients for each category	#Discussions for each category	#Total patients (%)	#Total discussions (%)
Thyroid	Non-rare disease	Borderline thyroid tumours	41	43	–	–	499 (92.7)	538 (89.1)
		Differentiated Thyroid carcinoma	457	494	1	1		
	Rare disease	Medullary thyroid carcinoma	22	36	7	13	39 (7.3)	66 (10.9)
		Poorly-differentiated thyroid carcinoma	1	1	–	–		
		Anaplastic thyroid carcinoma	6	13	–	–		
		Other^a^	3	3	–	–		
Parathyroid	Non-rare disease	Parathyroid adenoma	3	3	–	–	3 (75.0)	3 (75%)
	Rare disease	Parathyroid carcinoma	1	1	–	–	1 (25.0)	1 (25%)
Adrenal	Non-rare disease	Suspicious adrenal mass	28	32	1	4	128 (62.7)	165 (55.7)
		Benign adenoma	48	62	–	–		
		Atypic or lipid-poor adenoma	24	35	–	–		
		Adrenal hyperplasia	19	24	–	–		
		Myelolipoma	8	8	–	–		
	Rare disease	Pheochromocytoma	24	37	16	23	76 (37.3)	131 (44.3)
		Adrenocortical carcinoma	19	42	2	6		
		Metastases	8	8	–	–		
		Other^b^	7	15	–	–		
Paraganglioma	Rare disease	Head&Neck PGL	13	17	22	37	51 (100)	86 (100)
		Abdominal PGL	6	11	10	21		
Neuroendocrine neoplasm	Rare disease	GEP-NEN	30	38	1	1	38 (100)	48 (100)
		Lung carcinoids	7	9	–	–		
Total							835	1038

a, Two cases of lymphomas, one case of angiosarcoma; b, 6 cases of ganglioneuroma, five cases of schwannoma, one vascular cystic lesion, one renal oncocytoma and two adrenal oncocytoma;

N°, number; PGL, paraganglioma; GEP-NEN, gastroenteropancreatic neuroendocrine neoplasms.

**Table 3:** Logistic regression analysis considering the need for multiple discussion as readout.

Variable	Wald	p	OR	95CI (lower-upper)
**Gender**	0.000	1.000	1.000	0.662-1.510
**Age**	0.672	0.412	1.006	0.992-1.019
**Thyroid cancers**	0.362	0.548	0.798	0.382-1.666
**Adrenal masses**	**8.065**	**0.005**	**2.602**	**1.345-5.034**
**Familiar syndrome**	**8.674**	**0.003**	**2.995**	**1.443-6.217**
**Rare disease**	**22.928**	**<0.001**	**3.588**	**2.127-6.053**

OR, odds ratio; CI, confidence interval.Bold numbers highlight the statistical significance.

### 3.2 Board queries

**Figure 1:**
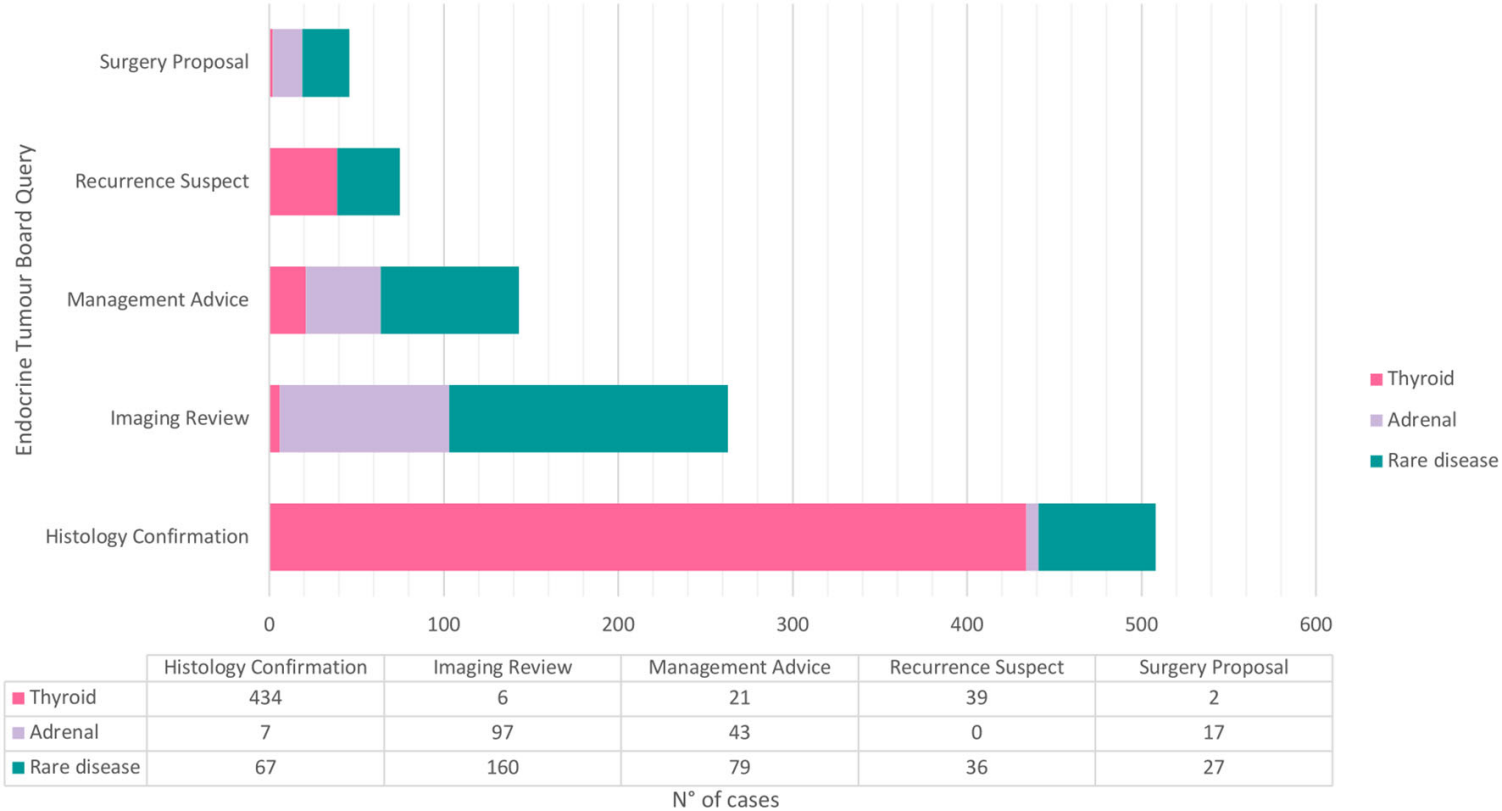
Graphic representation of the main Endocrine Multidisciplinary Tumour Board queries according to sporadic thyroid carcinomas, adreanal masses or rare disease. Three parathyroid adenomas were excluded for graphical purpose.

According to **Table 1**, the main EMTB queries were 509 (49%) histological confirmation, 263 (25.3%) radiological or (less frequently) nuclear medicine imaging reviews, 144 (13.9%) management advice, 75 (7.2%) recurrence suspect, and 47 (4.5%) surgery proposal. **Figure 1** shows the main EMTB queries according to sporadic thyroid cancer, adrenal masses, and rare endocrine tumours.

**Figure 2:**
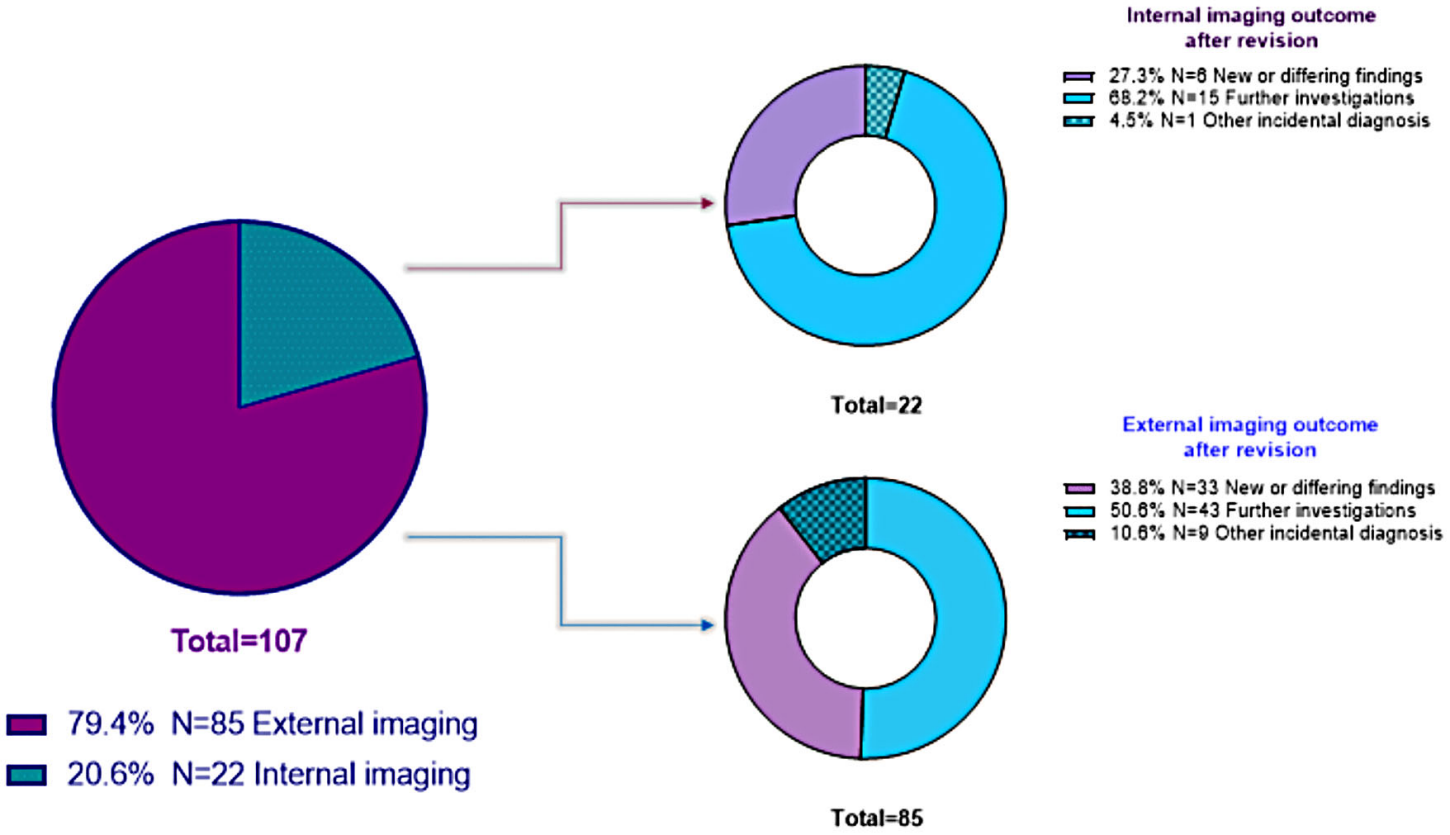
Graphic representation of re-evaluated reports after imaging revision, according to internal or external diagnostic.

In 730 (70.3%) cases, the diagnostic workup (histology or imaging investigations) was fully performed at Careggi Hospital, while in 308 (29.7%) cases, it was performed outside. Of 263 queries for imaging review, only 72 were related to internal imaging, while the majority of cases (n=191, 72.6%) were raised for imaging performed outside (p<0.001). In the latter cases, the EMTB conclusion was follow-up for 88 cases (46.1%) and surgical or medical treatments for 18 cases (9.4%), while for 85 cases (44.5%), the initial radiological report was overturned. **Table 4** shows the rate of internal and external cases discussed for imaging revision, according to each EMTB outcome. When outcome revision of internal (n=72) vs. external (n=191) diagnostics was compared, significant differences were found. Internal imaging cases had more often an indication to follow-up (59.7% vs. 46.1%, p=0.033, respectively), and the imaging report was less often re-evaluated (30.6% vs. 44.5%, p=0.027, respectively). No differences were found concerning the treatment indication rate (9.7 vs. 9.4%, p=0.553) (**Table 4**), and the need for imaging revision was independent of specific endocrine tumours (i.e., thyroid, adrenal, or rare cancers) (p=0.279). When adjusted for age, gender, and being a sporadic or familial rare disease (no/yes), having performed external radiological procedures (no/yes) was confirmed as the only risk factor for being classified as "re-evaluated" after EMTB discussion in a logistic regression analysis (OR=1.836, 95CI:1.022-3.298, p=0.042; **Table 5**). Reasons for the re-evaluation of imaging reports have been attributed to several issues (**Figure 2**). In half of the external cases (49.4%), new findings or differing results were observed at EMTB revision, including 10.6% of cases with an incidental diagnosis of another disease, and in about 51% of the cases, a further diagnostic was requested. The typology of diagnostic insights was histological re-challenge for 7% of cases, nuclear medicine imaging for 35%, and specific radiological imaging for 58%. Finally, for 10 of these cases, additional investigations were also suggested: five hormonal assessments, one genetic investigation, and four specialistic evaluations.

**Table 4:** The outcome of radiological and nuclear medicine review according to internal and external workup.

Outcome	Radiological procedures		p ^a^
	Internal	External	
**Follow-up**	43 (59.7%)	88 (46.1%)	**0.033**
**Treatment**	7 (9.7%)	18 (9.4%)	0.553
**Re-evaluated^b^**	22 (30.6%)	85 (44.5%)	**0.027**
**Total**	72 (100%)	191 (100%)	

^a^comparisons are performed according to internal vs. external diagnostic groups.

^b^re-evaluated means significantly different outcomes after Endocrine Multidisciplinary Tumour Board revision.Bold numbers highlight the statistical significance.

**Table 5:** Logistic regression analysis with the variable "re-evaluated case (no/yes)" as readout.

Variable	Wald	p	OR	95CI (lower-upper)
**External radiological procedures, yes**	**4.128**	**0.042**	**1.836**	**1.022-3.298**
**Age**	0.448	0.503	1.006	0.988-1.024
**Gender**	0.116	0.733	1.091	0.660-1.806
**Rare disease, yes**	0.663	0.416	0.801	0.470-1.366

Bold numbers highlight the statistical significance.

### 3.3 Additional recommendations and treatment strategies for EMTB

Low-risk differentiated thyroid carcinoma (DTC) or borderline thyroid tumours (n=325, 31.3%) have systematic follow-up indications and less often require additional EMTB recommendations (p<0.001). Of 713 remaining cases, 224 (31.4%) required specific diagnostic evaluations. The main further diagnostic investigations required upon EMTB discussions were radiological imaging with specific execution modalities (33.9%), nuclear medicine imaging (33.1%), clinical and molecular assessment (20.5%), or histological insight (i.e., re-biopsy or specific immunohistochemistry) (12.5%).

From the hormonal perspective, 444 cases were examined to verify whether or not they were associated with any hormonal secretion. Of those, EMTB required in 71 (15.9%) cases further hormonal investigations, including screening for hormonal secretion in 36 cases (50.7%), specific hormonal pre-surgical preparation in 27 cases (38.0%), and provocative diagnostic tests in 8 cases (11.2%).

Besides follow-up and further investigations (see above), in 267 cases (25.7%), the EMTB was able to provide therapeutic indications, including surgery for 118 cases (44.2%); radioiodine for DTC in 109 cases (40.8%); and systemic or loco-regional treatment for 26 (9.7%) and 17 cases (6.4%),respectively.

### 3.4 Case-control survival

Due to their aggressiveness and poorer prognosis, ATC and ACC patients from the present dataset have been selected for a sensitivity analysis regarding survival.

**Figure 3:**
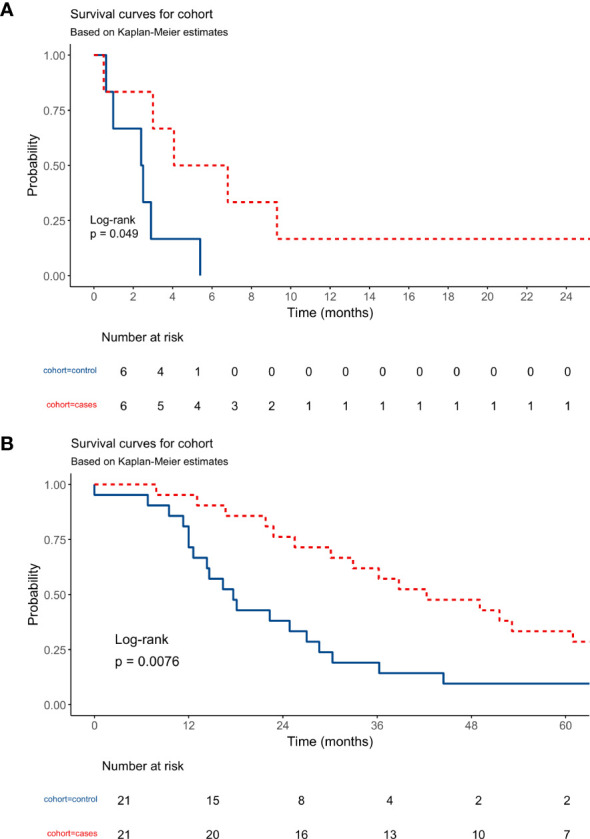
**(A)** Kaplan-Meier curves shows the overall survival of Anaplastic Thyroid Carcinoma patients from case versus control cohorts. **(B)** Kaplan-Meier curves shows the overall survival of Adrenocortical Carcinoma patients from case versus control cohorts.

According to the 8th edition of the American Joint Committee on Cancer (AJCC) AJCC/TNM cancer staging system, all ATC cases are, by definition, stage IV disease (13–15). To perform a survival comparison, a control group of six patients was randomly (the latest insertion in the database) selected from the ATC-TGCA population ("cBioPortal for Cancer Genomics," n.d.) and matched by age. The mean overall survival (OS) of the ATC cases subset was 7.84 (± 3.3 months, 95 CI:1.27-14.41 months) compared to 2.46 months (± 0.69 months, 95CI:1.10-3.82 months) of the control group, p=0.049. One patient from the cases' cohort was still alive at the end of this study. **Figure 3A** shows the Kaplan-Meier curves comparing the cases and control groups of ATC patients.

Considering ACC, the 21 cases were staged as follows: one patient at stage I (2.4%), nine at stage II (21.4%), three at stage III (7.1%), and eight at stage IV (19.0%), according to the 8th edition of the AJCC staging manual for ACC (13). A group of 21 controls was obtained as before and age- and stage-matched with cases for OS comparison ("cBioPortal for Cancer Genomics," n.d.). The mean OS of the cases subset was 51.92 (± 7.61 months, 95CI: 37.0-66.8 months) in contrast to 26.17 months (± 5.78 months, 95 CI: 14.84-37.5 months) of the control group, p=0.0076. 16 out of 21 patients (76.2%) of the cases cohort were still alive at the end of this study, including four patients with IV-stage disease. **Figure 3B** shows the Kaplan-Meier curves comparing the cases and control groups of ACC patients.

## 4 Discussion

The present study offers a tertiary centre perspective on the usefulness of the EMTB in managing endocrine cancers. Introducing a multidisciplinary approach has become pivotal in referral institutes to address advanced, complex, and rare tumours correctly (2). Indeed, multimodal decision-making is the favoured model to optimise patients' therapeutic pathways, which may require multiple EMTB discussions, as shown in the cases of familiar and rare endocrine cancers (OR=2.99, p=0.003 and OR=3.588, p<0.001, respectively). Present results show that internal radiological image revisions are critical to guarantee tailored patient care. Of note, a more comprehensive interpretation of patients' investigations often leads to reconsidering previous reports' conclusions in light of experts' imaging analysis and clinical, biochemical, and hormonal information. Indeed, when patients' imaging was derived from external centres, further investigations were more often required (p=0.027), independently from patients' age and gender or suffering from a rare disease (p=0.042). Finally, in line with MTBs of traditional oncology, rare and highly aggressive cancers showed improved OS when discussed within the EMTB.

Established in 2014, the Careggi EMTB has a long tradition of teamwork and serves as a referral centre for several rare diseases, having been involved in managing complex endocrine tumours. Currently, significant differences in EMTB composition, functions, and types of cases discussed still exist nationwide. This heterogeneity hinders the ability to compare different EMTB groups and compel a specific collaborative effort to standardise queries and functions of this board, enhancing patients' therapeutic opportunities. In this context, the present study aimed to systematise the EMTB purposes and highlight the peculiarities of this board from a real-life perspective.

In addition to previous statements, the present findings underscore some key points regarding the use of EMTB. First, due to the high rate of slow and long-surviving carcinomas, a skilled EMTB is essential at specific time points in these patients' histories. A particular example is the suspicion of iodine-refractoriness or disease progression in differentiated thyroid cancers (16). In these cases, owing to limited therapeutic resources, physicians must identify the optimal moment to adjust treatment strategies, prioritising the most beneficial therapy based on patients' histories. To achieve this, various physician perspectives must be integrated, and all clinical, biochemical, and hormonal information should be made accessible, exemplifying the case of EMTB. Second, rapidly evolving and aggressive cancers should always be assessed by EMTB and referral centres for pathology to positively impact survival (17). In the current population, these favourable results were noted for rare and lethal tumours like ATC and ACC patients. The former shows a longer survival, with one patient who is still alive at the end of this study, having a long-term favourable response to initial treatment. This aligns with a previous large ATC study (17) that demonstrated how multidisciplinary and multimodal approaches offered advantages for patient care. Overall, survival outcomes for ATC remain disappointing due to the very limited treatment options, yet new targeted therapies and insights into tumour biology motivate further steps in this direction (14, 18). Conversely, the ACC subgroup exhibits significantly better survival compared to the control group, with improved outcomes even at advanced stages. Once again, these results can be attributed to better and more rapid patient restaging, tailored therapeutic approaches, including local and systemic treatments, and access to clinical trials. Third, in the context of endocrine cancers, another major issue is hormonal secretions. Given their harmful nature, these conditions should be carefully addressed alongside disease recurrences. Indeed, undiagnosed hormonal secretions from Cushing's and Carcinoid syndromes or chromaffin disorders could lead to potential additional risks for patients; hence, they have to be diagnosed and treated to prevent acute and chronic complications. Of note, surgical or invasive procedures represent critical situations for these patients, as they may trigger random and uncontrolled hormonal secretions (i.e., carcinoid or hypertensive crisis for carcinoid disease and pheochromocytoma, respectively) (19, 20) or result in hypocortisolism crisis when removing cortisol-secreting masses without suitable preparation (21). The hormonal pitfalls may be overlooked in endocrine tumour care. As observed in our population, more than one-third should undergo specific hormonal preparation because of invasive procedures, but about 16% of cases still require further hormonal insights. The negative long-term outcome in patients improperly treated for tumour secretions has unveiled a growing interest in patients with the hormonal syndrome. For instance, in neuroendocrine tumours with carcinoid disease, the rates of carcinoid crisis have shown an increasing trend (19, 22, 23), and undertreated patients suffer from lower survival compared to non-secreting tumours (24). Alternatively, when suitably prepared with alpha-blockers, pheochromocytomas/paragangliomas undergoing surgical interventions experience a lower rate of intraoperative haemodynamic instability (25).

Out of the present experience, very few studies about the EMTB are available in the literature. Although the advantage in general management of patients is confirmed, major differences in population size, kinds of endocrine tumours, and designs prevent most of the comparisons with the present study. For instance, Savitz et al. (26) observed the importance of EMTB discussions in their decennial experience, reporting a variable rate of management change from 20% to 79%, according to a narrow subset of physicians interviewed by questionnaire (N=12). Although these authors analysed a smaller cohort (N=608) over ten years, including benign thyroid disease, they confirmed the need for multiple discussions for complex cases (36%) (26). Another study focused on MTB discussions for thyroid cancer (27) on a population of 284 patients over six years confirmed that 15% of cases required management change after discussions, and about 42% required additional imaging. Considering adrenal masses, Chiapponi et al. (28) evaluated a consecutive series of 100 adrenalectomies, observing significantly higher guidelines adherence after the MTB's discussions, with only 7% of cases undergoing unnecessary surgery for final benign diseases. Of note, most of the latter cases presented another concomitant extra-adrenal tumour. Thus, in doubtful cases, the authors suggested EMTB to discuss adrenal biopsy, a usually feared procedure for adrenal masses, due to potential hormonal adverse events or tumour spreading. However, the expertise of EMTB can more safely select the most suitable cases to perform adrenal biopsy to improve the surgical indication in this fringe.

Considering the current study, we must acknowledge several limitations. In particular, the retrospective and monocentric design limits our ability to establish causation or the effectiveness of EMTB discussions. This single-centre analysis may not be representative of all endocrine tumours, as some patients may have been managed outside the board. The lack of a comparison with an institution lacking EMTB is a significant limitation that prevents conclusions about the EMTB's impact on patient outcomes. Survival analyses have been conducted on two very rare and aggressive cancers but in a small subset of patients who were compared to external and anonymous control groups without information about management at their centres of origin. Nonetheless, this is the largest and most comprehensive study to date regarding the real-life use of an EMTB. The current results suggest that this board may enhance patient history in several ways: by providing a tailored definition of disease status, avoiding unnecessary treatments, prioritising imaging or hormonal insights, and, above all, improving survival rates for aggressive cancers. Establishing a uniform policy regarding the composition and functions of the EMTB is a step toward the future of care for endocrine tumour patients. For these reasons, this study also aimed to systematise the queries typically submitted to the team, promoting a shared understanding and consistent terminology for future comparisons among different team centres.

In conclusion, the experiences of traditional MTBs, such as lung and breast cancer ones, have already taught lessons of good clinical practice and benefits for patient care when using a comprehensive and complementary medical approach (1–4). To raise the standard of care for endocrine cancers, it is essential not only to build EMTBs but also to centralize patients suffering from rare and complex tumours to referral institutes because experience and expertise are two sides of the same coin.

## Data Availability

The datasets presented in this article are not readily available because Restrictions apply to the availability of some or all data generated or analysed during this study to preserve patient confidentiality or because they were used under license. The corresponding author will on request detail the restrictions and any conditions under which access to some data may be provided. Requests to access the datasets should be directed to luisa.petrone@unifi.it.
